# “They Know, They Agree, but They Don’t Do”- The Paradox of Tuberculosis Case Notification by Private Practitioners in Alappuzha District, Kerala, India

**DOI:** 10.1371/journal.pone.0123286

**Published:** 2015-04-24

**Authors:** Sairu Philip, Petros Isaakidis, Karuna D. Sagili, Asanarupillai Meharunnisa, Sunilkumar Mrithyunjayan, Ajay M. V. Kumar

**Affiliations:** 1 Government T.D. Medical College, Alappuzha, Kerala State, India; 2 Operational Research Unit, Médecins Sans Frontières, India; 3 International Union Against Tuberculosis and Lung Disease, South-East Asia Regional Office, New Delhi, India; 4 State TB Training and Demonstration Centre, State TB Cell, Directorate of Health Services, Thiruvananthapuram, Kerala, India; Universitat Autònoma de Barcelona. CIBERES, SPAIN

## Abstract

**Background:**

Despite being a recognized standard of tuberculosis (TB) care internationally, mandatory TB case notification brings forth challenges from the private sector. Only three TB cases were notified in 2013 by private practitioners compared to 2000 TB cases notified yearly from the public sector in Alappuzha district. The study objective was to explore the knowledge, opinion and barriers regarding TB Notification among private practitioners offering TB services in Alappuzha, Kerala state, India.

**Methods & Findings:**

This was a mixed-methods study with quantitative (survey) and qualitative components conducted between December 2013 and July 2014. The survey, using a structured questionnaire, among 169 private practitioners revealed that 88% were aware of mandatory notification. All patient-related details requested in the notification form (except government-issued identification number) were perceived to be important and easy to provide by more than 80% of practitioners. While more than 95% felt that notification should be mandatory, punitive action in case of failure to notify was considered unnecessary by almost two third. General practitioners (98%) were more likely to be aware of notification than specialists (84 %). (P<0.01). Qualitative purposive personal interviews (n=34) were carried out among private practitioners and public health providers. On thematic framework analysis of the responses, barriers to TB notification were grouped into three themes: ‘private provider misconceptions about notification’, ‘patient confidentiality, and stigma and discrimination ’and ‘lack of cohesion and coordination between public and private sector’. Private practitioners did not consider it necessary to notify TB cases treated with daily regimen.

**Conclusion:**

Communication strategies like training, timely dissemination of information of policy changes and one-to-one dialogue with private practitioners to dispel misconceptions may enhance TB notification. Trust building strategies like providing feedback about referred cases from private sector, health personnel visit or a liaison private doctor may ensure compliance to public health activities.

## Introduction

India, with 2.2 million cases annually, accounts for one-fourth of global incidence of tuberculosis (TB). In 2012, National Tuberculosis Programmes (NTPs) notified 6.1 million of the estimated total of 8.6 million TB patients to the World Health Organization (WHO) and 10–40% of notifications were from non-NTP care providers [1]. Of the estimated 2.9 million global missed cases, nearly one million TB cases were missed in India. These were patients who were either not diagnosed or not reported to NTPs. It is estimated that about 40% of TB patients in India are treated in the private health sector [2]. Public-Private sector collaboration in TB care has resulted in improvement in case notification and better treatment outcome [3–8].

The Government of India declared TB a notifiable disease in May 2012 and created a web-based, case-based notification system called NIKSHAY. It became mandatory that all public and private health providers notify TB cases to the designated public health authorities. Notification provided an opportunity to support the private sector in ensuring adherence to standards of TB care which included helping patients with right diagnosis, treatment, follow-up, contact tracing, linkages to social support systems and monitoring disease trends [9].

Kerala, a state in India, responded to TB Notification by registering 1221 hospitals, 454 laboratories and 1591 clinics from private sectors in NIKSHAY and notifying 430 TB cases from private sector in all districts during a one year period [10].

Alappuzha, a district in south Kerala with 2.1 million population [11] registers 2000 TB patients annually under NTP. Though 126 hospitals, 17 laboratories and 62 clinics from private sector were registered in Nikshay from Alappuzha, only three TB cases were notified in NIKSHAY web portal from the private sector from January to September 2013 [10].

Poor notification from private sector may be due to a variety of problems [12]. Identifying these hidden issues may help to address the problem effectively in a culturally sensitive manner. Hence, the specific objectives of the study were to assess the knowledge and opinion about TB notification, its process and to explore barriers in TB notification among general and specialist private practitioners offering TB services in Alappuzha.

## Materials and Methods

### Ethics

Ethics approval was received from the Institutional Ethics Committee of Government T.D.Medical College Alappuzha and the Ethics Advisory Group of International Union Against Tuberculosis and Lung Disease (The Union), Paris, France. Written informed consent was obtained from all study participants.

### Study design

This was a cross-sectional, mixed-methods study including a quantitative and a qualitative component and conducted between December 2013 and July 2014.

### Study Setting

Alappuzha, the smallest district of Kerala, has a population density of 1492 persons/sq.km. [13]. The District TB control program comprises of District TB Centre (DTC), Sub-district- TB Unit (TU), and Peripheral Health Institutions (PHIs) [14, 15]. There are four TUs in the district comprising of 18–23 PHIs. The private sector comprises of private clinics run by single practitioners and polyclinics or hospitals with multi-specialty services.

### Study population

#### Quantitative

The study population for the quantitative component included all private practitioners of Alappuzha treating presumptive TB patients. This included general practitioners who had a bachelor’s degree in medicine and specialists like physicians, pediatricians and pulmonologists. Surgeons, gynecologists etc were included only if they had a presumptive TB case during the past one year.

#### Qualitative

The private practitioners and public health providers interested to share their views on TB notification were purposively chosen for the qualitative component.

### Data collection

#### Quantitative

The list of private health institutions was obtained and the Personal Relations Officer was first contacted in each institution. A list of doctors in the institution who were eligible for the study was prepared with the administrator’s permission. The appointments were made at a convenient time for each doctor. Their knowledge and opinion regarding TB notification and its mechanism were recorded in a self-administered **structured questionnaire** completed within five to ten minutes under the guidance of the principal investigator. It included questions on opinions, perspectives and processes regarding mandatory notification. The responses were recorded using five point Likert item-type questions ranging from strongly agree to strongly disagree or by providing multiple choice options.

#### Qualitative

Personal interviews of 10 to 20 minutes were carried out at a time convenient to the doctor by the principal investigator who was formally trained in qualitative research methods. Since the private practitioners felt inhibited by audio recording, the personal interviews were recorded as field notes by the principal investigator. Where clarity was lacking, the notes were read and its meaning confirmed with the interviewee. The personal interviews were based on a topic guide which included questions related to their perception of tuberculosis as a public health problem, prevalent practices on managing a presumptive TB case and perceived barriers to TB notification.

### Data management and analysis

#### Quantitative

We used EpiData software for data entry and analysis (version 3.1 for entry and version 2.2.2.182 for analysis, EpiData Association, Odense, Denmark). Data were summarized as proportions and chi-square test was used for comparisons among different groups. A P value ≤ 0.05 was regarded as statistically significant.

#### Qualitative

The field notes were transcribed and manually coded by the principal investigator. Themes were subsequently reviewed and discussed with the team. Similar basic themes were grouped as organizing themes and then into a global theme, utilizing a thematic network analysis method as described by Attride-Stirling [16].

## Findings

### Quantitative

The quantitative survey included 169 private practitioners—from all tuberculosis units in Alappuzha district, the response rate being 80%.

The awareness of private practitioners regarding mandatory notification is summarized in Table 1.

**Table 1:** The proportion of private practitioners who had heard about mandatory TB Notification in Alappuzha district of Kerala, India.

Demographic variables	No:-	Heard about Mandatory Notification
		No (%)
Total respondents	169	149(88)
**Gender**		
Male	129	115(89)
Female	40	34(85)
**Age groups**		
less than 40 years	35	29(83)
40 to 55 years	46	41(89)
More than 55 years	88	79(90)
**Location**		
Urban	79	69(87)
Rural	90	80(89)
**Type of Practitioner**		
General	50	49(98)
Specialist	119	100(84)
**Type of Specialist**		
Physician	36	27(75)
Pediatrician	36	33(92)
Pulmonologist	7	7(100)
Surgeon	7	5(71)
Others	33	28(85)
**Years of experience**		
Less than 10 years	23	22(96)
10 to 20 years	31	24(77)
21 to 30 years	28	26(93)
31 to 40 years	53	48(91)
More than 40 years	34	29(85)

The overall proportion of practitioners who were aware of the mandatory TB notification system was 88%. General practitioners were significantly more likely to be aware about mandatory notification compared to specialists. (98% vs. 84%, p = 0.04). The notification included patient and disease related details. The opinion of the private practitioners regarding the importance and ease of provision of these minimum details are summarized in Table 2.

**Table 2:** The proportion of private practitioners who ‘Agreed or Strongly Agreed’ on the importance of and ease to provide the details for mandatory TB Notification from Alappuzha district of Kerala, India.

	General Practitioner	General Practitioner	Specialist	Specialist
	
	(N = 50)	(N = 50)	(N = 119)	(N = 119)
Details in TB	Important	Easy	Important	Easy
Notification form	No (%)	No (%)	No (%)	No (%)
Name	47(94)	43(86)	109(92)	107(90)
Father’s or husband’s name	43(86)	42(84)	97(82)	106(89)
Patient age	49(98)	43(86)	117(98)	112(94)
Patient's sex	43(86)	44(88)	102(86)	110(92)
Government issued identity Number	20(40)	20(40)	49(41)	56(47)
Address	50(100)	42(84)	117(98)	101(85)
Pin code	38(76)	35(70)	105(88)	91(76)
Phone number	45(90)	36(72)	109(92)	96(81)
Date of diagnosis	49(98)	40(80)	118(99)	98(83)
Date of treatment initiation	49(98)	40(80)	117(98)	95(80)
Site	50(100)	39(78)	115(97)	101(85)
Type of patient	50(100)	37(74)	117(98)	97(82)
Basis of Diagnosis	50(100)	39(78)	116(97)	97(82)
Treatment details	49(98)	39(78)	117(98)	102(86)

Among patient related details, government issued identity number was perceived to be the least important and least easy to provide. TB disease-related details were perceived to be important by 98% of the private practitioners and easy to provide by approximately 80%.

Perspectives regarding TB Notification among private practitioners are summarized in Table 3.

**Table 3:** Perspectives regarding mandatory TB Notification among private practitioners in in Alappuzha district of Kerala, India.

Perspectives regarding notification	General Practitioners	Specialists	P value
		
	N = 50	N = 119	
Supports private sector	44(88)	98(82)	0.49
Helps right diagnosis	46(92)	93(78)	0.04
Helps right treatment	48(96)	103(87)	0.09
Helps follow-up of patients	46(92)	103(87)	0.45
Helps tracing of contacts	46(92)	112(94)	0.86
Helps to find contacts for chemoprophylaxis	48(96)	105(88)	0.19
Helps in initiating community support systems	44(88)	94(79)	0.24

More general practitioners (92%) compared to specialists (78%) believed that notification helped in right diagnosis of TB (P = 0.04).

The sources of knowledge and preferences regarding TB Notification are summarized in Table 4.

**Table 4:** Sources of knowledge and preferences regarding mandatory TB Notification among private practitioners in Alappuzha district, Kerala, India.

Preference & Processes	General Practitioner	Specialist
	N = 50	N = 119
	No (%)	No (%)
**Sources of Knowledge regarding notification** *		
Government	37(76)**	65(65)**
Private	10(20)**	23(23)**
Others	11(22)**	22(22)**
**Notification** to be made mandatory	49(98)	112(94)
**Preferred methods of notification** *		
Post	7(14)	23(19)
Mobile	5(10)	28(24)
Email	15(30)	46(39)
Through health worker	32(64)	64(54)
Through own staff	0(0)	8(6)

*Multiple responses

**Out of those who have heard about notification

Preferred methods of notification were through health worker, email and mobile. Perceptions regarding government support and the actions taken in relation to mandatory TB Notification are summarized in Table 5.

**Table 5:** Perception of private practitioners regarding government support and the actions taken in relation to mandatory TB Notification in Alappuzha district, Kerala, India.

Governmental support and actions			
	General practitioner	Specialist	P value
	n = 50	n = 119	
	No (%)	No (%)	
**Present level of support from government**			
None	19(38)	63(53)	
Minimal	15(30)	24(20)	
Sufficient	12(24)	29(24)	
Strong	4(8)	3(3)	0.70
**Expected level of support from government**			
None	1(2)	7(6)	
Minimal	6(12)	11(9)	
Sufficient	23(46)	65(55)	
Strong	20(40)	36(30)	0.06
**Type of support expected from the government** *			
Training	20(40)	75(63)	0.002
Drugs to be made available	25(52)	89(75)	0.04
Health worker visit	28(56)	92(77)	0.007
Feedback of patients	34(68)	96(81)	0.14
**Private Practitioners view of giving "nil report" for TB**			
Unnecessary	9(18)	37(31)	
No opinion	16(32)	34(29)	
Necessary	25(50)	48(40)	0.22
**Action taken by government in case of "failure to notify'**			
Unnecessary	11(22)	36(30)	
No opinion	17(34)	42(35)	
Necessary	22(44)	41(34)	0.45

*Multiple responses

Eighty five percent of private practitioners expected sufficient or strong support from the government. Feedback of TB cases referred by private practitioners to the public system was the most common support expected by them. Significantly higher number of specialists expected support in the form of free TB drugs (P = .007), health worker visit to their institution (P = 0.04) and training (P = 0.002), compared to non-specialists.

### Qualitative

Personal interviews were taken from 34 participants (specialists = 20, general practitioners = 6, government personnel and administrator = 8, refusal = 2).

**Fig 1:**
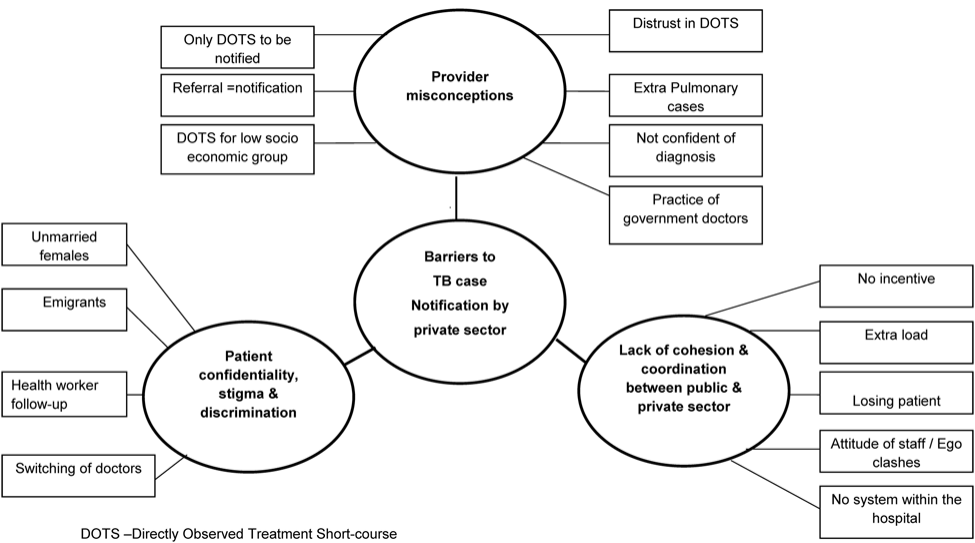
Thematic Analysis showing “Barriers to Tuberculosis Notification by Private Sector” in Alappuzha district, Kerala, India.

We constructed a thematic network organizing the basic and organizing themes around the global theme “Barriers to notification’ and represented it as a web-like, non-hierarchical figure (Fig 1).

### Barriers to notification

The global theme “Barriers to notification” had three organizing themes, namely “Provider misconceptions regarding non-DOTS regimens,” “Patient confidentiality, stigma and discrimination” and” lack of cohesion and coordination between the public and private sector”.

#### Provider misconceptions regarding non-DOTS regimens

Most of the private practitioners referred TB cases especially sputum smear-positive cases to the nearby government centre for Directly Observed Treatment Short course (DOTS).

*“I refer all my cases to the government*. *We are so busy*. *The government is doing a good job ensuring treatment*. *The patient gets free drugs*. *Why deprive them of it”*

*(General practitioner, 47y, M).*

Some did not notify because they thought patients on the national treatment protocol (intermittent, thrice-weekly DOTS) only need to be reported.

*“All my cases on DOTS are reported*. *Oh*! *I did not know that those on other regimes need to be reported to government*.*”*

*(Pediatrician, 50y, F).*

Some felt that DOTS is meant for the low socioeconomic status.

*“DOTS is for those who cannot afford”*

*(Physician, 59y, M).*

Subscription to non-DOTS regimen was a reason for not notifying. One specialist had distrust in DOTS.

.*“In DOTS the possibility of relapse is high and the clearance is late*. *So I prefer daily regime”*

*(Pulmonologist, 47y, F).*

One preferred daily treatment to NTP’s thrice-weekly intermittent regimen for specific cases.

*“I get pleural effusion and extra pulmonary cases*. *In my experience*, *daily regimens work better than DOTS (thrice-weekly regimens)*. *I have been working here over 30 years*. *I am like a family doctor*. *I am confident that patients will not default*. *I am sensitive to my patients needs”*

*(Physician, 59y, M).*

Private practitioners did not notify when they were not certain of their diagnosis.

*“Some cases are not notified if the doctor is not sure of his diagnosis and has started the drugs as a trial”*

*(Medical Officer, Tuberculosis Unit, 46y, M).*

Government doctors who practice privately sometimes subscribe non-DOTS regimes which they do not notify. This conveys the message to the private practitioners that they need not notify non-DOTS regimes.

*“AKT4 (a commercially available anti-TB drug kit) is widely prescribed by doctors*. *For pediatric patients*, *syrup is preferred.*.. *[…]*. *Even some government doctors in their private practice prescribe AKT4 and they do not notify…..it is difficult to address this”*

*(Senior Treatment Supervisor, 35y, F, NTP* ***).***

#### Patient confidentiality, stigma and discrimination

Tuberculosis cases were not notified to the government when issues related to confidentiality discrimination or stigma occurred as in case of unmarried females.

*“Due to ………her father begged not to send his daughter to the government or report her diagnosis to anyone*. *I knew the family for a long time*. *I thought I will ensure treatment and its completion*. *I didn’t notify as I had to respect his wish”*

*(Physician, 54y, M).*

Doctors did not notify in case of emigrants who could not stay locally till treatment completion.

*“They come home on leave for two months*. *Diagnosis of TB makes them anxious*. *They need to go back on time*. *They promise to continue treatment*. *They are afraid that by staying back to complete treatment or making the diagnosis known may jeopardize their job*. *These cases may not be notified”*

*(Physician 62 M).*

The higher socioeconomic groups were concerned with breach of confidentiality by health workers.

*“The upper and middle socioeconomic groups come to the private hospital*. *They do not want the health worker to locate their house and come for follow up*. *It arouses curiosity of neighbors*. *They do not like it*. *They feel the health worker may reveal the diagnosis to somebody they are familiar with*.*”*

*(Physician, 65y, M)*

Another issue was “switching of doctors” on being diagnosed to have TB.

*“Some people cannot believe they got TB*. *They want to confirm diagnosis with another doctor*. *So they stop coming to me*. *And I lose contact”*

*(Pulmonologist, 43y, M).*

#### Lack of cohesion and coordination between public and private sector

There is no system in private hospitals that keeps all doctors informed about notification.

*“When we get cases*, *we do not know-whom to report*, *how to report and where to report*?*”*

*(Physician, 55y, M)*

Record keeping is considered as an extra load when staff and time is less and there are no incentives for notifying disease.

*“If DOT workers do the paperwork of non-DOTS regime*, *it will be good”*

*(Pulmonologist, 43y, M).*

When patients are referred to government from private sector, some consider it as ‘losing’ patients.

*“When private hospitals refer patients to government*, *it is like losing patients*.*”*

*(Medical Officer, Tuberculosis Unit, 38y, M).*

Ego clashes occur between the government and private personnel.

*“Approach to private by government is very bad*. *They do not give us respect*.*”*

*(General Practitioner, 48y, F).*

*“I do not like the step motherly attitude of the government to private”*

*(Physician, 59y, M)*

A specialist felt that a patient put on daily regimen was unnecessarily switched over to DOTS by government health worker.

*“……the patient was on daily regimen*. *Some health worker confused him and put him on DOTS*. *He came back to us with persisting post operative sinus*. *Put him on AKT4 and quinolones and resolved”*

*(specialist, 42y, M).*

There was need for coordination even within the public health sector—between the general health service staff and TB staff to support the private sector.

*“It is difficult for us to know the new private clinics of 20 to 23 Primary Health Centers (PHC)*. *It is best known to the health worker who looks after one sub centre*. *If there is coordination between us*, *it is easy*. *But all PHC’s do not give priority to TB”*

*(Senior Treatment Supervisor, 47y, M).*

## Discussion

Our study revealed that the awareness regarding mandatory TB case notification among private practitioners was high in Alappuzha district in Kerala. The TB related details in notification form was agreed to be important and easy to provide by 80% of private practitioners. This is a paradox considering the poor TB notification from the private sector in this district. The paradox is therefore a “perception-practice gap.” TB suspects approaching the private practitioners and put on intermittent regimens (DOTS) were referred to the government to be notified through the public system. Thus a certain number of patients approaching the private sector were being reported through the government sector. This raises the pertinent question as to whether there are cases that are not notified.

The qualitative data filled in the details not captured by the quantitative data. Some private practitioners felt that notification meant “referral of TB cases to government “or that “only patients put on DOTS need to be reported”. Thus the cases on non-DOTS regimes are missed. Some practitioners were hesitant to notify non-DOTS regimes because they felt DOTS was the only treatment accepted by the government programme. The confidentiality issues in notification point towards the underlying stigma prevalent in the community. The high proportion of private practitioners not favoring “nil reporting” and “punitive action for non-notification” could have these underlying issues.

The real purpose of notification has not been clearly understood by the private sector. There is need for the NTP to build a trustful relationship with the private sector. Notification should be perceived as an essential tool for assessing the burden of TB in the community rather than one to audit the private sector. The government personnel should not be judgmental towards the private practitioner. Changes in the programme, treatment protocols and rationale for the same need to be disseminated timely with the private practitioners in a platform of mutual respect and cooperation.

The quantitative study revealed a subtle difference between the general practitioners and specialists. General practitioners due to their contact with the health workers were comparatively more conversant with government programmes. A significant number of general practitioners (92%) thought that notification helped in right diagnosis compared to specialists (78%). Specialists have asked for support like drugs, health worker visit and training significantly more than general practitioners. This point to the need of NTP to reach out to specialists, who may otherwise remain unaware due to their physical inaccessibility to health workers consequent to patient load and specialist status. The strategies for addressing the barriers in TB notification are summarized in Table 6.

**Table 6:** Possible suggestions for communication and trust building in private sector to address barriers in TB Notification.

Communication and trust building in private sector- suggestions.
**Related to notification process**
-Notification form to contain relevant and minimum personal and TB related Information
-Smart reporting of TB cases through newer mobile/net applications
- Include additional options for private sector e.g.:-“referred as possible case” or “referred to public sector for notification”
-Identify reporting process which assures confidentiality of patients
**Related to ‘Provider misconceptions about non-DOTS regimens'**
- Training programmes with focus on role of notification in TB control and relevance of details in notification form
- Establish personal contact and platform to clarify technical doubts of private Practitioners
- Timely Information to private sector about policy changes
- Government doctors in private practice to conform to national treatment protocols
**Related to ‘Lack of cohesion and coordination between public and private sector’**
- Feedback to private sector for cases referred to public sector
- Recognition measures like non monetary incentives, certification
- Motivated liaison officer for private public partnership at district/state level
- Coordination between TB staff and PHC staff through regular review meetings.
**Related to ‘Patient confidentiality, stigma and discrimination’**
- Stigma addressed through media campaigns highlighting issues e.g.;-stigma of TB among unmarried females.
- Flexibility in programme in case of special cases e.g.; emigrants
-Confidence building of doctors regarding confidentiality of notified cases
- Soft skill training of government staff to address confidentiality issues &promote professionalism.
-Motivational and attitude building training for health care personnel
- Leadership training for self awareness/interpersonal relation/overcoming ego/conflict resolution/teamwork to be included in medical curriculum

Notification brings similar challenges in high burden regions of the world. The common reasons for not reporting communicable diseases by private doctors in Taiwan were “violation of privacy of patient”, “troublesome reporting procedures’, “absence of a reward”, or “no penalty for non reporting’ [17].

It was realized a decade ago that inclusion of private practitioners could increase case detection and notification of TB. [18]. In spite of innumerable efforts for public private partnership in tuberculosis care, barriers to the same hold true for notification namely “inadequate training and lack of information”, “not remunerative”, “technical doubts about the programme”, “liaison and interaction challenges” in this sector [18].

Improvement of interpersonal skills of health providers involved in DOTS programme was recommended in relation to utilization of TB care in India [19]. Strategic efforts are required to ensure consistent motivational and attitudinal building for personnel involved in health care services (both private and public) to ensure compliance in a public health measure like notification. Perceptional conflicts influence private-public divide in national programmes [20]. Models in Pakistan, India and Nepal reiterate the untapped but significant potential of private sector to impact public health programmes [3, 4, 21]. A standardized reporting system yielding a comprehensive epidemiological situation of tuberculosis may evolve only over many years of collaboration like the European TB Surveillance System [22].

The study has several limitations due to the limited geographical area the data were drawn from. Since the private sector is largely unorganized, small number of new clinics might have been missed in spite of efforts to locate all of them. However we believe that our study, using mixed-methods, revealed important insights and produced evidence which can be extrapolated and expanded to other parts of the country and may be the region. A single investigator interviewing all the subjects minimized inter-observer bias. The reporting of study adhered to STROBE and COREQ guidelines [23, 24].

## Conclusion

Notification of tuberculosis as a public health measure for control of tuberculosis needs to be showcased to the private practitioners in India. They may be encouraged to report all cases treated. Specialists in private hospitals should be targeted as important stakeholders in TB notification. Communication and trust building strategies for behavioral changes in notification in private sector should include flexible changes in notification process, feedback of referred patients, timely dissemination of policy changes, soft skills training for government health personnel and involvement of a liaison officer dedicated to public-private coordination.

## Supporting Information

S1 Data Sheet (ZIP)
